# Supercritical carbon dioxide extraction of lipids and carotenoids from *Rhodotorula toruloides* CBS 14 in comparison with conventional extraction methods

**DOI:** 10.1186/s13068-025-02632-7

**Published:** 2025-03-21

**Authors:** Yashaswini Nagavara Nagaraj, Johanna Blomqvist, Sabine Sampels, Jana Pickova, Mats Sandgren, Peter Gajdoš, Milan Čertík, Volkmar Passoth

**Affiliations:** 1 Department of Molecular Science, Swedish University of Agricultural Sciences, Uppsala BioCentre, P.O. Box 7051, 750 07 Uppsala, Sweden; 2 Institute of Biotechnology, Faculty of Chemical and Food Technology, Slovak University of Technology, Radlinského 9, 812 37 Bratislava, Slovakia

**Keywords:** *R. toruloides* CBS 14, Supercritical carbon dioxide extraction, Lipids, Carotenoids, Folch method, Acetone extraction, Saponification

## Abstract

**Background:**

Oil from oleaginous yeasts has the potential to replace non-sustainable vegetable oil as raw material to produce food, feed, biofuels, or biochemicals. Co-produced compounds like carotenoids may be helpful to obtain economically viable bioprocesses. Identifying appropriate extraction methods is a bottleneck both for establishing oleaginous yeasts as cell factories for both oil and carotenoids production and for analysis of intracellular compounds like lipids and carotenoids. We conducted extractions using supercritical carbon dioxide (SC-CO_2_) and conventional solvent methods to extract and analyze lipids and carotenoids from *R. toruloides* CBS 14 cells grown on wheat straw hydrolysate. The lipid extracts were analyzed using gas chromatography (GC), and the carotenoids were identified and quantified using ultra-high-performance liquid chromatography (UHPLC).

**Results:**

Four main carotenoids in the extracts from both extraction methods were identified including β-carotene, γ-carotene, torularhodin, and torulene. Interestingly, torularhodin was the major carotenoid extracted using SC-CO_2_ extraction, followed by torulene. This was different from the conventional acetone extraction method, where β-carotene was the main carotenoid. After the conventional extraction, torularhodin and torulene underwent degradation due to the saponification step, which was necessary to remove lipids before UHPLC analysis. The total carotenoid concentration obtained from SC-CO_2_ extraction was 332.09 ± 27.32 μg/g dry weight compared to 19.9 ± 2.74 μg/g dry weight in acetone extraction. A small amount of carotenoids was observed to be lost into the lipid extract, but this loss was not as substantial as that seen with acetone extraction. Additionally, the total lipid content in samples extracted using SC-CO_2_ was significantly lower than that obtained using the conventional Folch method. GC analysis revealed that oleic acid was the major fatty acid in both lipid extracts, followed by palmitic acid and linoleic acid. Notably, the proportion of unsaturated fatty acids was higher in the extracts from the SC-CO_2_ method compared to the conventional method.

**Conclusion:**

These findings indicate that the SC-CO_2_ extraction method outperformed conventional methods by preserving the integrity of unsaturated lipids and retaining an abundance of carotenoids, resulting in high-quality extracts.

## Introduction

In recent years, the consumption of fats and oils as food ingredients has significantly increased, constituting more than 80% of their overall usage [1]. Lipids play a vital role in cooking, baking, and food preparation. Additionally, they find application as animal feeds, providing improved nutritional value and energy density to livestock diets [2]. As the demand continues to rise, alternative sources for plant oils are being explored. One focus has been targeted on microbial oil (MO) as sustainable fat source. MO is synthesized and accumulated by specific microorganisms, known as oleaginous microorganisms, through cultivation processes that utilize renewable resources as substrates [3]. Lignocellulosic biomass presents a promising substrate due to its renewability and abundant nature, featuring substantial quantities of readily usable sugars [4, 5]. Many oleaginous yeasts possess the capability to convert sugars, polysaccharides, glycerol, and other compounds into oil [3]. The major constituents of the produced lipids are primarily neutral triacylglycerols (TAGs), which contain energy-rich fatty acids resembling the fatty acid composition of vegetable oil and therefore have the potential to be utilized in the production of food, feed, biofuels, and various biochemicals [3, 6]. Some oleaginous yeasts can also produce carotenoids as secondary metabolites [7–9]. Carotenoids are used as natural colorants and antioxidants in the food industry to enhance the visual appeal of products [10]. They are also employed as dietary supplements, either in their natural form or as purified extracts, due to their antioxidant and health-promoting properties [11].

To fully utilize the biotechnological potential of oleaginous yeasts for the production of lipids and carotenoids, it is crucial to develop effective extraction processes. This is because the lipids and carotenoids produced by these yeast cells are sensitive to peroxidation which can be induced by for example heat and light [12]. Therefore, cautious extraction methods need to be adopted to ensure efficient recovery of these valuable compounds preserving their quality and bioactivity to ensure the full benefit of oleaginous yeasts for the production of high-value lipids and carotenoids [13, 14].

Traditionally, the lipids and carotenoids have been extracted from oleaginous yeasts using organic solvents. However, this conventional method has limitations due to the significant use of harmful solvents, multiple extraction steps, and reduced effectiveness caused by the barriers presented by cell envelopes [15–17]. To overcome these challenges, various physical techniques such as heat treatment, ultrasound, high-pressure homogenization, bead-milling, and vigorous shaking have been employed before or during extraction [18]. These procedures aim to disrupt the cell envelopes, allowing solvents to penetrate the cells and solubilize the lipids and carotenoids, thereby improving the extraction yield [16, 19]. These physical approaches enhance the accessibility of solvents to intracellular components, leading to more efficient extraction processes with reduced solvent consumption and increased recovery of lipids and carotenoids [18].

SC-CO_2_ extraction has emerged as a gentle technique for extracting natural substances aimed at applications in the food and pharmaceutical sector [20]. Although supercritical fluid extraction (SFE) has been recognized for its solvent properties for over a century, its commercial application remained unfeasible due to limited focused research in this area [21]. However, continuous advancements in extraction technologies have elevated SFE to a new level, enabling lipid and carotenoid extraction using supercritical fluids such as carbon dioxide [22, 23]. These fluids are characterized by operating above the critical temperature and pressure of the compound, resulting in specific properties [24]. In the supercritical state, carbon dioxide exhibits unique properties including high compressibility, liquid-like density, enhanced diffusivity, low viscosity, and low surface tension, similar to an organic solvent.

The properties of supercritical fluids, particularly SC-CO_2_, play a significant role in their ability to permeate and extract compounds from matrices compared to conventional organic solvents [16]. Researchers have indicated that, compared to conventional organic solvents, SC-CO_2_ exhibits higher diffusivity and lower density, viscosity, and surface tension. Additionally, these properties can be widely adjusted by altering the operational conditions [25, 26]. These characteristics enable SC-CO_2_ to penetrate both micro and macro porous components, allowing for selective extraction, fractionation, and purification of target compounds [25]. SC-CO_2_ extraction offers several advantages over traditional methods. Firstly, it is non-toxic and eliminates the need for potentially harmful organic solvents, making it a safer option for both operators and consumers [27, 28]. This quality renders it suitable for food and feed-related applications. It also preserves the integrity of the extracted compounds, as it operates under milder conditions, minimizing thermal degradation [29–31]. Researchers have focused on optimizing the extraction process by adjusting key parameters such as temperature, pressure, extraction time, and CO_2_ flow rate. By fine-tuning these variables, optimal extraction conditions can be achieved, ensuring maximum yield and quality of the extracted natural products [32].

Today, SC-CO_2_ is widely employed in extracting valuable oils from high-fat-content nuts such as almonds [33], peanuts [34], and walnuts [35]. It is also used to obtain food-grade oils like grape seed oil [36] and pumpkin seed oil [37]. Furthermore, SC-CO_2_ has been successfully applied for carotenoid and chlorophyll extraction from the microalga, *Nannochloropsis gaditana*, using methanol [38]. However, research on the application of SC-CO_2_ for extracting lipids and carotenoids from oleaginous yeasts remains limited.

The objective of this study was to evaluate the effectiveness of supercritical carbon dioxide (SC-CO_2_) extraction for recovering lipids and carotenoids from an oleaginous red yeast, cultivated on lignocellulose hydrolysate. By establishing SC-CO_2_ extraction, the research sought to establish a more sustainable, efficient, and high-yield method for obtaining these valuable compounds from oleaginous yeasts. The advantageous acquaintance of this work is not only evaluating the impact of SC-CO_2_ extraction but also providing a valuable comparison with conventional extraction methods.

## Materials and methods

### *Rhodotorula toruloides* cultivation

#### Strain maintenance and inoculum preparation

*R. toruloides* CBS 14 was obtained from the Westerdijk Fungal Biodiversity Institute, Utrecht, the Netherlands. Cells were stored in frozen stocks at − 80 °C. The inoculum was prepared as described before by Nagaraj et al*.* [8]. Briefly, cells from YPD-agar plates (glucose 20 g/L, peptone 20 g/L, yeast extract 10 g/L, agar 15 g/L) were inoculated into 300 mL of YPD (glucose 20 g/L, peptone 20 g/L, yeast extract 10 g/L) in a 3 L Erlenmeyer flask and incubated at 25 °C for 48–72 h at 150 rpm to produce sufficient quantity of viable cells to be able to inoculate the bioreactor cultivation to an initial OD of 5. The cells were harvested by centrifugation (4000*g*, 10 min), washed twice with sterile saline solution (NaCl, 9 g/L), resuspended in saline, and inoculated into the fermenters. All chemicals were purchased from Sigma-Aldrich (Europe) unless otherwise stated.

#### Bioreactor cultivation

The cells from the initial inoculum were added to 1.5 L of growth medium in Minifors 2, Bench-Top bioreactors (INFORS HT, Switzerland, working volume 2 L). The yeast cells were cultivated in the bioreactors as described previously [8]. In brief, the prepared yeast inoculum was introduced to nitrogen-limited growth medium within the bioreactors, containing filter-sterilized wheat straw hydrolysate and 1.7 g/L YNB (yeast nitrogen base without amino acids and ammonium sulfate), 2 g/L (NH_4_)_2_SO_4_, 7 g/L KH_2_PO_4_, 2 g/L NaH_2_PO_4_, 1.5 g/L MgSO_4_·7H_2_O, and 1 g/L yeast extract. Immediately after inoculation, 100 mL of culture broth was collected for determining optical density and cell dry weight. After 96 h, when all the sugar was consumed, the fermentation was stopped, and the cells were harvested, washed, French-pressed (Constant Systems LTD, Daventry, UK) at 40 kPa and at − 5 °C, freeze-dried, and stored at -20 °C until further processing.

### Extraction methods

#### Conventional solvent extraction

##### Lipid extraction by Folch method

Lipids from the yeast cells were extracted using the Folch method [39] with some modifications as explained previously [8]. All analyses were performed in triplicates. In brief, freeze-dried cells underwent lipid extraction through sequential treatments involving 1 M HCl, 0.8% KCl, Folch solution (chloroform: methanol, 2:1 v/v ratio), and pure chloroform. The cell material was heated in the HCl at 75 °C for 1 h to enable a more effective cell disruption [8]. The separation was executed using a separatory funnel, with the lower lipid-rich phase collected in a pre-weighed glass tube. Subsequently, the glass tube containing the lipid phase was subjected to nitrogen gas to remove chloroform through evaporation. The resulting tube containing the lipids was then weighed. Finally, the dried lipid samples were resuspended in 1 mL hexane and stored at − 20 °C until further processing for methylation.

##### Carotenoid extraction using acetone

The carotenoid extractions were carried out in dark conditions by using the method described by Reif et al. [40] with some modifications as explained in detail in our previous work [8]. The extractions were performed in triplicates. In summary, French-pressed yeast biomass was subjected for the acetone extraction process and at the end of the extraction, the acetone collected in the glass tube was evaporated under nitrogen, leaving behind the carotenoid extract, which was further subjected to saponification using ethanol, butylated hydroxytoluene (BHT, 0.2 mg/mL in methanol), methanol, and methanolic potassium hydroxide (2 M KOH in methanol). This saponification was done to remove the lipids from the extracts, as they could interfere with the UHPLC analysis. The final saponified carotenoid extracts were diluted with methanol: acetone (1:1, v/v) before further analysis.

#### Supercritical carbon dioxide (SC-CO_2_) extraction

All the extractions were carried out in dark conditions using a supercritical extractor (Jasco Supercritical Extractor, SFE 4000 series, Kovalent AB, Italy), with freeze-dried biomass of *R. toruloides* CBS 14 loaded into the extraction vessel (10 mL capacity with possibility to extract 1–5 g of biomass) mixed with a layer of silica beads (SiLibeads Typ ZSA 2.2–2.5 mm) serving as solvent flow distributors. The extractor was equipped with a high-pressure modifier pump and a backpressure regulator for a stable and trouble-free constant flow of CO_2_. The fractions were collected in the automatic fraction collectors. The entire extraction process was controlled and operated using the software ChromNAV, version 2.3C for precise monitoring and regulation. All the extractions were made in triplicates.

##### Lipid extraction

The lipids from the freeze-dried biomass were extracted using a similar method as described by Milanesio et al*.* [41] with some modifications. The freeze-dried yeast cells were mixed with silica beads (yeast cell: beads, 3:2, w/w) and loaded into the extraction vessel. Extraction pressure was 300 bar, at a temperature of 45 °C and with a CO_2_ flow rate of 2 mL/min. The total extraction time was 180 min. Subsequently, the residue was subjected for carotenoid extraction. The lipids were collected in pre-weighed brown bottles and then dissolved in 1 mL hexane and stored at − 20 °C until further analysis.

##### Carotenoid extraction

Once the lipid extraction using pure SC-CO_2_ was completed, the carotenoids were extracted from the biomass extraction using a method similar to that described by Lim et al*.* [42] with some modifications. Ethanol (99.5% v/v) was used as a co-solvent in the process. The carotenoid extraction process was conducted at a pressure of 300 bar, a temperature of 50 °C, a CO_2_ flow rate of 2 mL/min, and a co-solvent flow rate of 0.2 mL/min. The extraction duration was set for a total of 180 min. To maintain the integrity of the extracted compounds, the resulting extracts dissolved in ethanol were stored in brown bottles at a temperature of − 20 °C until further analysis.

### Analyses

#### Fatty acid analysis

The extracted lipid samples were methylated, using the procedure with boron trifluoride reagent described previously by Nagaraj et al. [8]. Further, the methylated fatty acids were analyzed in a GC system (CP-3800, CTC Analytics AG, Switzerland) equipped with a split injector and a flame ionization detector and fitted with a 50 m long × 22 mm i.d., 0.25 μm film thickness, BPX 70 fused-silica capillary column. The GC was programmed to start at 158 °C, with temperature increasing at a rate of 2 °C min^−1^ until 220 °C and a final constant time of 13 min at 220 °C. The peaks were identified by comparing their retention times with those of the standard mixture GLC-68D (Nu-Chek Prep, Elysian, MN, USA) and other authentic standards. The response factors were also evaluated by comparison with the GLC-68D standard. All samples were applied in triplicates.

#### Carotenoid profile analysis in lipids using HPLC–DAD

Pigments were analyzed by HPLC. Analysis was carried out with an HP 1100 chromatograph (Agilent) equipped with a DAD. Pigment extracts in hexane (10 µL) were injected onto a LiChrospher^®^ 100 RP-18 (5 µm) column (Merck). The samples were separated by an elution system at a flow rate of 1 mL/min. The elution system was composed of solvents A, acetonitrile/water/formic acid 86:10:4 (v/v/v), and B, ethyl acetate/formic acid 96:4 (v/v), with a gradient of 100% A at 0 min, 100% B at 20 min, and 100% A at 30 min [43]. The carotenoid content was expressed as μg of β-carotene equivalent (β-EQ)/g of dried yeast weight.

#### Carotenoid profile in carotenoid extract analysis using UHPLC-PDA

Carotenoid composition analysis was performed using a Shimadzu UHPLC-Nexera instrument from Kyoto, Japan. The instrument setup included an autosampler (SIL-20AC), quaternary pumps (LC-20AD), a column oven (CTO-20AC), and a PDA detector (Shimadzu, model SPD-M20A) connected in series. LabSolutions software was employed for instrument control, data acquisition, and data processing. Carotenoid separation was accomplished using an analytical RP C18 Kinetex 100 column (100 mm length, 4.6 mm internal diameter, 2.6 μm particle size; Phenomenex) with a binary gradient system. The mobile phase A consisted of a mixture of acetonitrile and methanol in a ratio of 7:3 (v/v), while the mobile phase B comprised ultrapure water with 0.1% formic acid. The gradient elution program was as follows: 0–3 min, 60% B; 3–7 min, 100% B; 7–30 min, 100% B; and 30–35 min, 60% B. The flow rate was set at 0.3 mL/min, the column temperature was maintained at 40 °C, and a sample volume of 20 μL was injected. UV–visible spectra were acquired in the range of 250–600 nm using the PDA detector. Specific wavelengths were selected for the detection of individual carotenoids after screening of commercial standards (company) for the max absorption: β-carotene and torularhodin were detected at 450 nm, γ-carotene at 462 nm, and torulene at 478 nm [8]. The analysis was made in triplicates.

### Statistical analysis

Experiments were performed in triplicate, and the presented results are mean ± standard deviation. Student’s *t* test was performed to evaluate the significance of differences between the mean values using RStudio (Version 2023.06.1 + 524) software package. The differences were considered significant at *p* ≤ 0.05.

## Results and discussion

### Impact of extraction methods on yeast lipid recovery

**Fig. 1:**
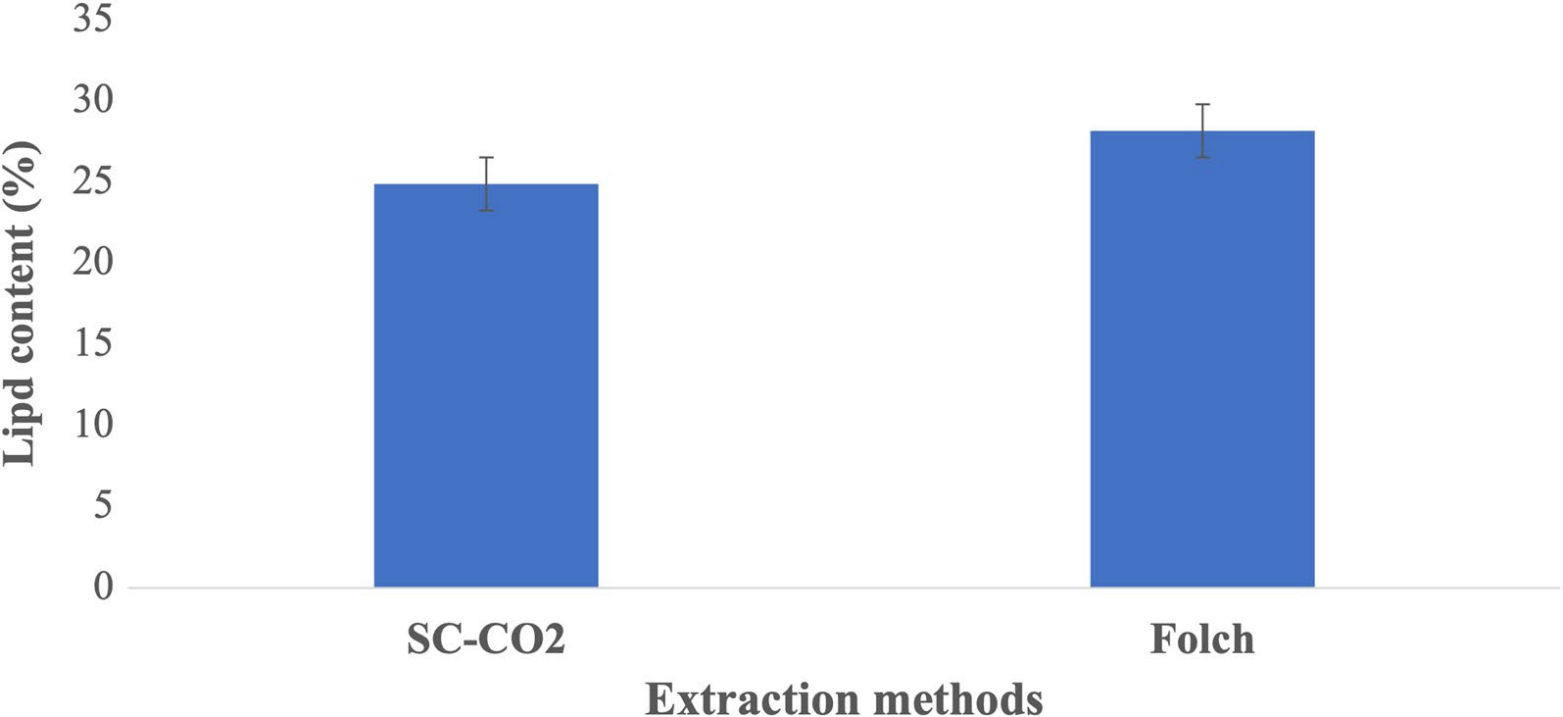
Average lipid content in dry cells of *R. toruloides* CBS 14 after extraction by Folch and SC-CO_2_ methods (*n* = 3). The difference in the extracted amount of lipid was significant (*p* < 0.05). Data presented as mean ± standard deviation

Lipid extraction from the yeast samples was performed using the Folch method and SC-CO_2_ extraction (Fig. 1). The results depicted in the figure indicate that the lipid content in the samples extracted with SC-CO_2_ was significantly lower (*p* < 0.05). Similar results have been found by Milanesio et al. [41] when extracting lipids from the yeast *Yarrowia lipolytica* with different methods. They compared Soxhlet and accelerated solvent extraction (ASE) using a mixture of chloroform and methanol in the same ratio as Folch and SC-CO_2_ extraction with and without ethanol. They found significantly lower lipid yield with SC-CO_2_ and also showed that pretreatment of the yeast mass with ethanol increased extraction capacity of SC-CO_2_.

Another aspect of lipid yield by different extraction methods is the polarity of the used solvents. Duarte et al*.* [44] highlighted that SC-CO_2_ extraction will not yield the certain fractions of lipids, namely, the waxes, phospholipids, sterols, and pigments, and hence result in a lower lipid recovery. In contrast, in Folch method, a mixture of polar and non-polar solvents improves extraction of polar lipids [45] and thereby enhances the lipid yield as earlier seen in algae [15, 46]. This argument is strengthened by the results of Milanesio et al*.* [41], showing a better extraction with a polar co-solvent added to the non-polar SC-CO_2_.

In the present study using ethanol as a co-solvent for lipid extraction was not an option as we wanted to separate the carotenoids from the lipids which would have co-eluted when using ethanol from the start of extraction.

### Fatty acid profile analysis and comparison of extraction methods

The lipid profiles of the two different extracts are presented in Table 1. The analysis showed that oleic acid (C18:1(n-9)) was the predominant fatty acid in the lipid extracts obtained from both extraction methods. Following oleic acid, palmitic acid (C16:0) and linoleic acid (C18:2(n-6)) were found to be the major fatty acids in the yeast lipids. This general composition is in line with our and others' earlier findings [8, 47, 48]. Moreover, the presence and amounts of linoleic acid and linolenic acid (C18:3(n-3)) were in line with our earlier results being a significant proportion of polyunsaturated fatty acids (PUFAs) in the yeast lipids. The composition and ratio of saturated to unsaturated fatty acids (SFAs/UFAs) are crucial indicators for assessing the nutritional and functional properties of oils [32]. In this study, the SFA/UFA ratios were determined for the extracts obtained through the Folch and SC-CO_2_ methods, resulting in ratios of 0.48 and 0.34, respectively. SC-CO_2_ extraction method resulted in lipid extracts with a significantly higher content of UFAs, accounting for approximately 70% of the total fatty acids, compared to the Folch method resulting in 62.3%.

**Table 1:** Quantification of different fatty acids in *R. toruloides* CBS 14 samples extracted using Folch and SC-CO_2_ methods (*n* = 3)

	Fatty acid profile (%) of the total fatty acids	
	Folch method	SC-CO_2_ method
C14:0	1.46 ± 0.08	1.18 ± 0.09
C16:0	23.8 ± 0.63	19.2* ± 0.76
C18:0	4.56 ± 0.29	3.31* ± 0.22
C24:0	0.38 ± 0.03	0.22* ± 0.02
C18:1 (n-9)	46.6 ± 1.55	50.9* ± 1.39
C18:2 (n-6)	14.0 ± 1.49	17.2* ± 1.33
C18:3 (n-3)	1.69 ± 0.24	1.85 ± 0.34
Total SFA	30.2 ± 0.55	23.9* ± 0.48
Total UFA	62.3 ± 0.70	70.0* ± 0.67
SFA/UFA	0.48	0.34

Data are presented as mean % ± standard deviation. Asterisks indicate statistically significant differences (*p* ≤ 0.05)

*SFA* saturated fatty acids, *UFA* unsaturated fatty acids

This difference is most probably connected to the above-described difference in lipid recovery. As mentioned, the polarity of the solvent will affect the composition of extracted lipids. To explore these effects further, there is a need to separate and quantify the lipid classes from the extracts and determine the fatty acid composition of the fractions.

Another aspect is the temperature. In the employed Folch method, the cells are treated with HCl at 75 °C for 1 h. This elevated temperature can lead to thermal degradation of PUFAs, leading to a reduction in their abundance, ultimately affecting the overall fatty acid composition of the lipid extract [32, 49]. In addition, also the carotenoids, which can act as antioxidants and protect the PUFAs from oxidative degradation, are sensitive to heat [50, 51]. If they are oxidized during the extraction process their antioxidative capacity, protecting the PUFAs is lost, most probably leading to an increased oxidation of PUFAs. In contrast, the SC-CO_2_ extraction method operates at lower temperatures, which helps minimize the thermal damage inflicted on UFAs and PUFAs present in the oil. Also, in SC-CO_2_ extraction, the process is conducted in the absence of oxygen, which further contributes to the protection of bioactive compounds from oxidation [32, 52]. Han et al*.* [32] achieved comparable outcomes, wherein they conducted a comparative analysis of the lipid composition of *Berberis dasystachya* Maxim. seed oil extracted by both SC-CO_2_ method and the conventional organic solvent method (using petroleum ether extraction). Their findings indicated that the SC-CO_2_ approach yielded greater proportions of UFAs and PUFAs when contrasted with the organic solvent method. Findings from Kayathi et al*.* [53] also confirmed that the lipid extracted from mango kernel using SC-CO_2_ was particularly rich in UFAs.

### *Analysis of lipid extract from SC-CO*_*2*_* for carotenoid profile*

**Fig. 2:**
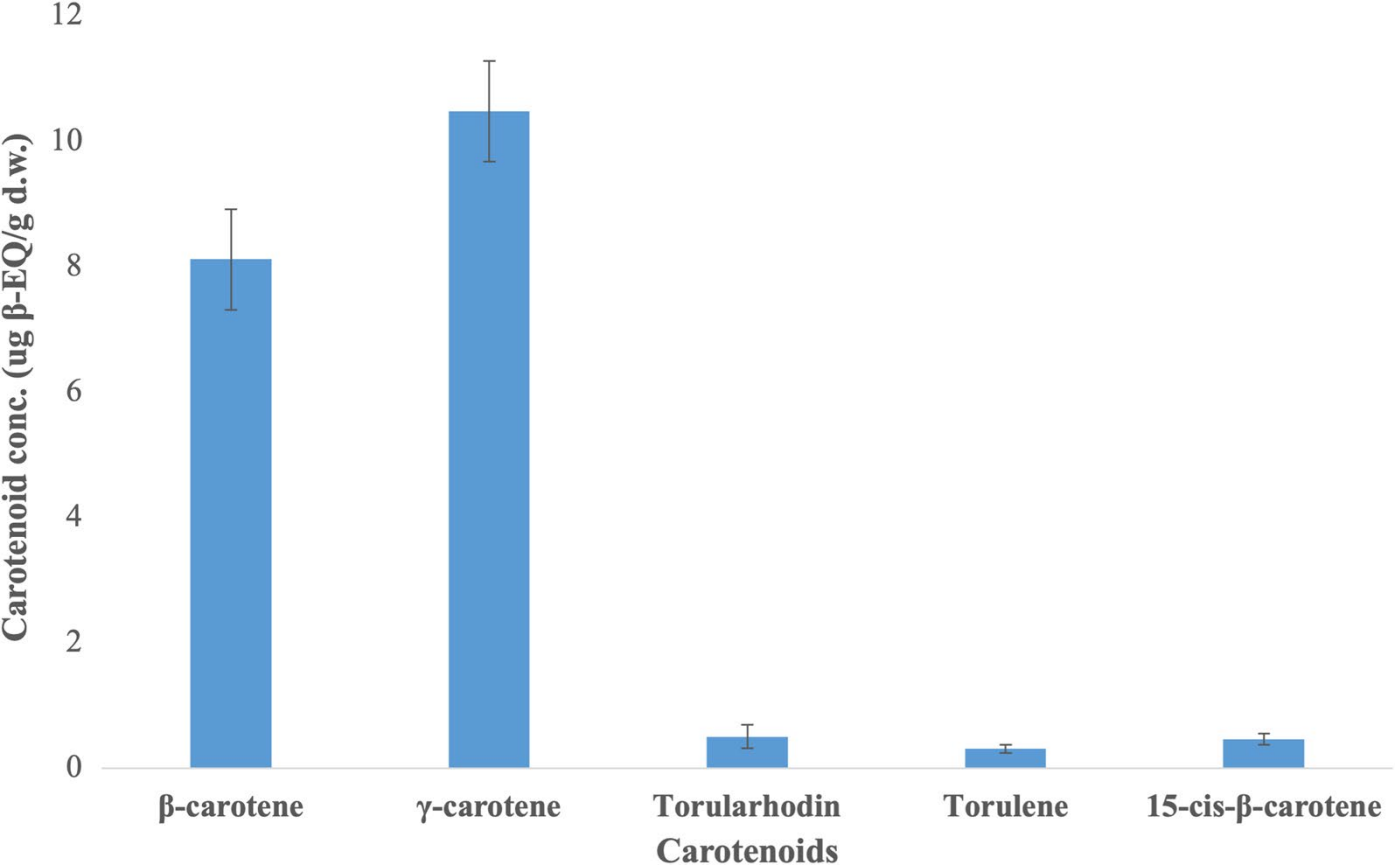
Individual carotenoid concentration in the lipid extract of *R. toruloides* CBS 14 as analyzed by HPLC–DAD (*n* = 3). Data presented as mean ± standard deviation

The extracted lipids using SC-CO_2_ were found to contain some carotenoids as well. These carotenoids were profiled using HPLC–DAD, revealing detectable amounts of β- and γ-carotene in the lipid extract (Fig. 2). Additionally, trace amounts of 15-cis-β-carotene, torulene and torularhodin were also present. A total of 19.85 ± 4.64 μg β-EQ/g d.w. carotenoid content was identified in the lipid extract. The presence of carotenoids in the lipid extract can be attributed to the non-polar, lipophilic nature of SC-CO_2_, which makes it an effective solvent for extracting low-polarity compounds like β- and γ-carotene and also contrasts with the lower solubility of torulene and torularhodin in SC-CO_2_ [23, 54, 55]. The use of co-solvents can enhance the solubility of target compounds and improve extraction selectivity [26]. As a result, when using pure SC-CO_2_, some amount of β- and γ-carotene are co-extracted along with the lipids.

### *Carotenoid analysis of the carotenoid extract from SC-CO*_*2*_

After the lipid extraction, the lipid-free samples were then subjected to extract the carotenoids produced in *R. toruloides* CBS 14 using the mixture of carbon dioxide and ethanol. The inclusion of ethanol to SC-CO_2_ has been found to enhance the solvent effectiveness of the supercritical fluid. This leads to the swelling of the sample matrix, causing an increase in internal volume and surface area. It therefore contributes to the decomposition of the cellular wall, potentially improving the bioavailability of carotenoids during the extraction process [50]. Additionally, by introducing ethanol, the polarity of CO_2_ can be modified, leading to enhanced solubility of carotenoids within the supercritical environment [16, 56, 57]. The obtained carotenoid extracts were further analyzed using UHPLC-PDA. The quantities of the four major carotenoids identified are presented in Table 2: β-carotene, γ-carotene, torulene, and torularhodin. With the SC-CO_2_ extraction torularhodin was found to be the most abundant carotenoid, followed by torulene.

**Table 2:** Quantification of individual carotenoids in *R. toruloides* CBS 14 extracted using SC-CO_2_ and acetone extraction methods

Carotenoids	Quantity of carotenoids (μg/g d.w.)	
	SC-CO_2_ method	Acetone extraction method*
β-carotene	25.4 ± 4.41	14.8 ± 0.28
γ-carotene	0.99 ± 0.11	4.20 ± 0.59
Torulene	105 ± 13.15	< LOQ
Torularhodin	200 ± 12.94	0.90 ± 0.28
Total carotenoids	332 ± 27.32	19.9 ± 2.74

Data are presented as mean ± standard deviation of carotenoid content in the samples from all three fermenters; Asterisks indicate data taken from previous work [8]

*d.w.* dry weight, *LOQ* limit of quantification

In a previous experiment, using acetone extraction method, β-carotene was showing the highest proportion in *R. toruloides* CBS 14 [8]. However, we have argued that torulene and torularhodin were not detected most likely caused by degradation during the saponification step employed (Table 2). The saponification step was crucial for eliminating unwanted lipids from the carotenoid extracts before UHPLC analyses. This resulted in a two-fold reduction of the total carotenoid content of the saponified carotenoid extract when compared to the unsaponified samples [8], strengthening our hypothesis that torulene and torularhodin were degraded, as those carotenoids have been found in *R. toruloides* earlier [47].

In contrast, the SC-CO_2_ method involved the extraction of lipids from the sample prior to carotenoid extraction. Consequently, the carotenoid extracts obtained were free from lipids, eliminating the need for saponification. These lipid-free extracts were directly injected into the UHPLC system. Torularhodin and torulene were observed in even higher concentrations than β-carotene, confirming our previous hypothesis that torularhodin and torulene were earlier degraded during saponification. This could also be seen in an increased amount of extracted total carotenoids with SC-CO_2_ extraction. In a study conducted by Martinez et al*.* [16] similar results were obtained, where upon SC-CO_2_ extraction of carotenoids from *Rhodotorula glutinis* with ethanol as the co-solvent, torularhodin was identified as the primary carotenoid. This finding is consistent with other studies that have also confirmed torularhodin as the most abundant carotenoid in various *Rhodotorula* species, accounting for approximately 60–70% of the total carotenoids extracted [58, 59]. Also, Hosseini et al*.* [60] confirmed enhanced carotenoid recovery from *Dunaliella salin*a extracts using SC-CO_2_ extraction, compared to the conventional extraction method (Soxhlet method). However, there might still be a variation in the content of these carotenoids, depending on strain and substrate as Qi et al*.* [47] found more than 10 times higher values of both total carotenoids in different strains of *R. toruloides*, grown on tea waste. Zheng et al*.* [48] on the other hand found β-carotene as the major carotenoid in different *R. toruloides* strains grown on cane molasses.

Although some carotenoid loss into the lipid extract occurred with SC-CO_2_ extraction, it was less substantial compared to the loss observed with acetone extraction. This suggests that SC-CO_2_ extraction offers a more effective recovery of carotenoids than acetone extraction.

## Conclusion

SC-CO_2_ extraction emerged as preferable method to extract high amounts of carotenoids and increased proportion of UFA from *R. toruloides*. Gas chromatography analysis showed that lipids extracted using SC-CO_2_ exhibited higher levels of UFA compared to the lipids extracted using the Folch method. SC-CO_2_ extraction revealed that torularhodin and torulene were the major carotenoids in *R. toruloides* CBS14. This finding can be of significance since these carotenoids are of high potential value since they have a higher antioxidant potential than β- and γ-carotene. Up to now, natural sources for their isolation are limited; thus, the established extraction may enable future biotechnological production of these carotenoids. The SC-CO_2_ extraction method demonstrated the ability to preserve the integrity of unsaturated lipids and abundance of carotenoids, resulting in high-quality extracts. However, it is important to note that despite the lower lipid recovery, the SC-CO_2_ extraction method offers the advantage of extraction without any organic solvents, eliminating the need for potentially harmful and environmentally unfriendly solvents. These findings hold great potential for future industrial applications and can serve as a starting point for further research in this area.

## Data Availability

No datasets were generated or analysed during the current study.

## Abbreviations

GC: Gas chromatography
HPLC: High pressure liquid chromatography
DAD: Diode array detector
PDA: Photodiode array
PUFAs: Polyunsaturated fatty acids
SFAs: Saturated fatty acids
UFAs: Unsaturated fatty acids
SC-CO_2_: Supercritical carbon dioxide
SFE: Supercritical fluid extraction
TAGs: Triacylglycerols
UHPLC: Ultra-high-pressure liquid chromatography
